# Hyaluronic Acid-Modified Nanoplatforms as a Vector for Targeted Delivery of Autophagy-Related Gene to the Endometriotic Lesions in Mice

**DOI:** 10.3389/fbioe.2022.918368

**Published:** 2022-07-01

**Authors:** Mengdan Zhao, Meng Zhang, Qin Yu, Weidong Fei, Tiantian Li, Libo Zhu, Yao Yao, Caihong Zheng, Xinmei Zhang

**Affiliations:** Women’s Hospital, School of Medicine, Zhejiang University, Hangzhou, China

**Keywords:** gene, nanoparticles, autophagy, endometriosis, targeted therapy

## Abstract

This investigation probed endometriosis treatment using targeted nanoparticles (NPs) to modulate autophagic activity. To that end, a novel form of polymer-based NP gene delivery platform consisting of polyethyleneimine (PEI) conjugated to stearic acid (SA) and nucleotides (DNA/siRNAs) and enclosed by hyaluronic acid (HA) was prepared. CD44 is highly upregulated in cystic lesions, and HA–CD44 binding in this specific nanoplatform was used to achieve targeted drug delivery to CD44-expression endometriotic tissues. The expression of autophagy-related genes was modulated to explore the importance of this process in the development of endometriosis. By inducing autophagic activity, we were able to reduce the size of endometriotic cysts and suppress the development of ectopic endometrium. To further confirm the relationship between autophagic activity and this disease in humans and animals, numbers of autophagic vesicles and autophagic protein expression were assessed in lesion tissue samples from patients, revealing there may be consistency between animal and human data. Overall, these data revealed the ability of this (PEI–SA/DNA) HA gene delivery system to regulate autophagic activity and, thereby, aid in the treatment of endometriosis.

## Introduction

Endometriosis is a serious condition wherein endometrial tissue is present outside of the uterine cavity, leading to a detriment in patients’ quality of life and mental health (Hung et al., 2021). Endometriosis is prevalent within over 1 in 10 women of childbearing age (Liu et al., 2021), and its incidence rates continue to increase annually. The primary symptoms of endometriosis include pain and infertility (Zhang et al., 2021). While a benign disease, endometriotic lesions exhibit malignant characteristics including the potential to invade other tissues, metastasize, and recur, making it a highly intractable and debilitating reproductive disease (Wang et al., 2020). Currently, the precise etiology of endometriosis remains unclear (Marquardt et al., 2021), with symptomatic surgical or drug-based treatment primarily being employed to treat affected patients. Although endometriosis is not fatal, an estimated 40% of patients will experience recurrent endometriosis within a 5-year period. Moreover, 1% of patients will develop a form of ovarian cancer as a consequence of the malignant transformation of these lesions (Mechsner, 2016; Kurose et al., 2021). It is, thus, important that the mechanistic basis for endometriosis be more fully clarified in order to guide the cost-effective treatment of these patients and eliminate associated clinical risks.

The retrograde menstruation implantation theory of endometriosis is widely accepted (Yang et al., 2017), but fails to explain why 90% of women of reproductive age exhibit retrograde menstruation, whereas just 3.7% suffer from endometriosis, nor does it account for endometriosis outside of the pelvic cavity. As such, other factors are also likely to contribute to the pathogenesis of endometriosis, with prior reports suggesting that genetic, epigenetic, and immunological factors can all shape endometriotic progression (Mehdizadehkashi et al., 2017; Lin et al., 2020). A range of molecular factors is thought to shape the development of endometriosis, including hormone signaling, microenvironmental changes, and abnormal immunological activity (Gadducci et al., 2021).

Autophagy is a conserved mechanism whereby cells are able to degrade and recycle damaged organelles and various macromolecular structures, and it plays a central role in many cancers and other tumor-like disease states (Ogasawara et al., 2020; Dong et al., 2021). Core autophagy-related proteins (Atg3, Atg5, Atg7, and Atg13) and other autophagy-associated proteins including Beclin-1 and Uvrag can inhibit the onset and progression of certain tumors (Wei et al., 2014; Lucas et al., 2020). Found that the low expression rate of Beclin-1 has a close correlation with the increase of the malignant degree of endometrial disease, as the expression of Beclin-1 protein from high to low was normal endometrium, endometrial hyperplasia, and endometrioid cancer (the expression in endometrial adenocarcinoma was the lowest among endometrial cancer although there is currently no research on the mechanism of different types of endometrioid carcinoma), thus indicating that the loss of Beclin-1 protein expression may be an early molecular event in the precancerous lesions of endometrioid carcinoma Zhao et al. (2006). Given its ability to inhibit tumor-like malignant behaviors, autophagy is also likely to prevent the pathogenesis of endometriosis (Yang et al., 2019; Kiyama, 2020). In some reports, reduced autophagic activity has been reported to inhibit endometriosis, although further research is necessary to clarify whether such activity is affected by periodic endometrial changes (Guo and Yu, 2019). As such, additional studies of the regulatory relationship between autophagy and endometriosis are warranted in an effort to guide the development of novel therapeutic approaches to treating this debilitating condition.

Beclin-1 is an essential autophagy-associated regulatory protein (Liang et al., 1999). Bcl-2 and other proteins could bind onto Beclin-1 BH3 domain, thereby shaping its ability to regulate autophagic activity. Relative Beclin-1 and Bcl-2 binding can thus be analyzed to gauge autophagy levels within cells. Binding between Beclin-1 and Bcl-2 inhibits autophagy, whereas free Beclin-1 can promote autophagic induction (Pattingre et al., 2005), and those compounds disrupt Bcl-2 binding to Beclin-1 or that promote Beclin-1 upregulation can induce autophagy. Potential approaches to achieving Beclin-1 upregulation include the exogenous delivery of various nucleic acids including DNA, mRNA, siRNAs, miRNAs, or antisense oligonucleotides (Yin et al., 2014). Gene therapy-based therapeutic areas are an area of active research interest, with several such products having received clinical approval to date (Mohammad, 2021). In the present study, a nanotechnology-based approach was employed to facilitate Beclin-1 gene delivery (White et al., 2015), thereby inducing Beclin-1 overexpression and enhancing autophagic activity in an effort to decrease the progression of endometriosis.

**FIGURE 1:**
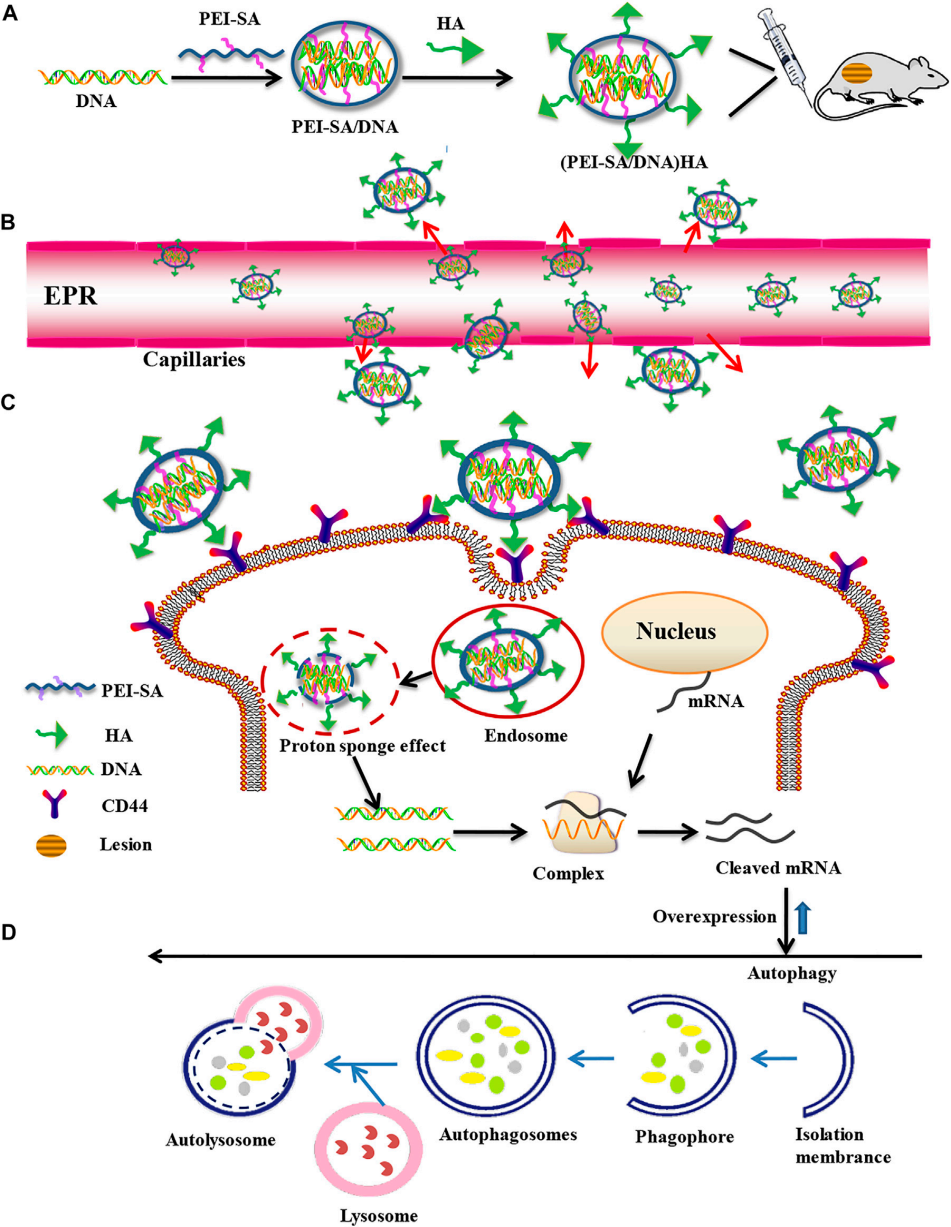
Construction of a targeted nanogene delivery system and key mechanisms of autophagy regulation *in vivo*. **(A)** Synthesis process of nanogene delivery system. **(B)** Uptake processes in the blood vessels. **(C)** Cellular uptake process. **(D)** Overexpression and autophagy processes.

We have previously successfully synthesized polyethyleneimine (PEI) and stearic acid (SA) conjugates (PEI–SA) by leveraging a chemical reaction for the -NH_2_ group of PEI and the -COOH group in SA, and such a resultant nanoplatform was employed as a drug delivery tool to treat rats harboring ovarian model tumors (Zhao et al., 2019). In the present report, we, therefore, employed a modified form of PEI–SA as a delivery carrier to achieve Beclin-1 overexpression. In an effort to minimize the systemic toxicity associated with these positively charged nanoparticles (NPs) and to improve their targeted activity, hyaluronic acid (HA) was employed for nanoparticle surface modification owing to its strong negative charge and ability to target CD44. The resultant nanoplatform was referred to as (PEI–SA/DNA) HA and its internalization, transfection efficacy, biosafety, and autophagy-inducing capabilities in endometrial cells were subsequently assessed. In addition, Beclin-1-conjugated (PEI–SA/DNA) HA and associated *in vivo* autophagic activity levels were assessed. Overall, we found that (PEI–SA/DNA) HA was readily endocytosed by endometrial cells, facilitating their efficient transfection and thereby enhancing autophagy and inhibiting endometriotic pathogenesis and progression in a murine model system following *in vivo* Beclin-1 delivery with negligible toxicity. In addition, siRNA delivery was used to achieve autophagic gene silencing in these model systems. Together, these results revealed that autophagy was able to effectively inhibit endometriosis, providing a novel approach to autophagy-focused therapeutic interventions for individuals suffering from endometriosis (a schematic diagram is shown in Figure 1).

## Materials and Methods

### Materials

Polyethylenimine (branched PEI, MW: 800 Da), ethidium bromide, and fluorescein isothiocyanate (FITC) were obtained from Sigma-Aldrich Chemical Co.™, Ltd., United States SA was procured through Shanghai Chemical Reagent™ (China). 2, 4, 6-trinitrobenzene sulfonic acid (TNBS) and 3-(4, 5-dimethyl-thiazol-2-yl)-2,5-diphenyl-tetrazolium bromide (MTT) were from Sigma Chemical Co.™ (MO, United States)> HA was obtained through MedChemexpress™, China. 1-Ethyl-3-(3-dimethylaminopropyl) carbodiimide (EDC) was procured through Medpep™, China. Trypsin, Dulbecco’s modified Eagle’s medium (DMEM), and trypsin-EDTA were procured through Gibco™ (United States). Fetal bovine serum (FBS) was from Sijiqing Biologic™, China. pcDNA3.1-Beclin1 was synthesized by GeneChem Co., Ltd., China, while Beclin1-siRNA duplexes were synthesized by Genepharma Co., Ltd., China. Mouse monoclonal anti-CD44 (EPR18668) was obtained from Abcam (United Kingdom). DAB/HRP/Blocking Buffer (DS9800) was from Leica (Germany). Additional reagents had analytical grades and were employed without requiring prior purifying protocols.

### Cell Culture

The Women’s Hospital School of Medicine Zhejiang University ethics committee allowed all human experiments within this present study (Approval No. 20160114), with all participants having provided informed consent to participate. Forty patients diagnosed with ovarian endometriosis, uterine leiomyoma, and infertility and who underwent laparoscopic surgery were recruited. In accordance with the findings of the surgery, the patients were divided into two groups, namely, the endometriosis (*n* = 20) and the control (*n* = 20) groups. All patients had a regular menstrual cycle, and none had undergone hormonal therapy within the last 3 months. We also excluded patients who suffered from other gynecological or autoimmune diseases. The tissues were cut into 1 mm pieces after washing thrice with PBS and digested with 0.1% type I collagenase at 37°C for 60 min. Cells were then passed through a 100 μm nylon cell filter followed by a 40 μm nylon cell filter, enabling the separation of epithelial cells from mesenchymal cells, the latter of which can pass through these filters to yield primary control endometrial stromal cells. This same approach was additionally used to process ovarian endometriosis tissue samples collected from endometriosis patients, thereby yielding primary ectopic endometrial stromal cells. Patients included in the present study experienced normal menstrual cycles and had not undergone hormone-based treatment within 3 months prior to surgery. After isolation, primary endometrial stromal cells were grown within DMEM/F 12 medium augmented by 12% FBS.

### Endometriosis Mouse Model Establishment

Ethical policies and protocols approved by the Institutional Animal Care and Use Committee of Zhejiang University Animal Center were used for this present study (Approval No. ZJU20160192). In total, 18 female BALB/c murines (6–8 weeks old) were employed for founding an endometriosis model as previously described by Li et al. (2020). Briefly, mice were subjected to ovariectomy and treated with estradiol benzoate (500 ng/murine/5 days, s.c.) 7 days prior to inducing endometriosis. Then six mice were selected at random to serve as donors, with removed uterine horns being collected and transferred within sterile normal (0.9%) saline. These uterine horns were then split longitudinally and macerated into minute (<1 mm^3^) segments using scissors, with endometrial fragments from individual murines being suspended within 1.2 ml sterile 0.9% saline and introduced (i. p.) within two recipient murines. At 24 h following endometriosis induction, murines were randomized into three groups (*n* = 4/group). This same modeling approach was additionally used with six BALB/c nude mice for *in vivo* fluorescence microscopic imaging.

### Polyethyleneimine–Stearic Acid Synthesis

The PEI–SA copolymer was synthesized employing an optimized version of a previously published protocol (Zhao et al., 2019). In brief, 100 mg SA was dissolved within 25 ml of hot ethanol, while 250 mg of PEI was dissolved within 20 ml of distilled water. These two solutions were then combined together at 80°C with constant stirring. Next, 375 mg of EDC were introduced to the resultant mixture and stirred for 24 h at 300 rpm. This mixture was consequently dialyzed against 10% ethanol for 48 h through an appropriate dialysis membrane (MWCO: 3.5 KDa, Spectrum Laboratories™, United States) for removing by-products, followed by dialysis for 2 h against distilled water. The resultant dialysate was then lyophilized to yield PEI–SA.

### (Polyethyleneimine–Stearic Acid/DNA) HA Preparation and Characterization

Different PEI-SA solutions were purified using a 0.22 μm millipore filter. An appropriate volume of PEI-SA solution and specific siRNA or DNA samples were then mixed for 30 s *via* vortexing, and stable PEI-SA/DNA NPs were prepared *via* electrostatic interaction. The resultant PEI-SA/DNA particles were coated with HA to yield (PEI-SA/DNA) HA.

The mean hydrodynamic diameter and zeta potential values for (PEI-SA/DNA) HA were determined with a Zetasizer instrument (3000HS, Malvern Instruments™, United Kingdom). Transmission electron microscopy (TEM, JEOL, JEM2100, Japan) was used to assess (PEI-SA) HA and (PEI-SA/DNA) HA NPs morphological characteristics. The resultant degree of amino substitution (SD) was determined *via* the TNBS method as reported previously (Zhao et al., 2012).

### Gel Retardation Assay

Varying PE–SA concentrations were combined with DNA (1 μg DNA) with gentle vortexing, and were allowed to stand at ambient temperature for 30 min pre-analysis. Electrophoresis was consequently conducted (100 V, 25 min) within TAE buffer [40 mM Tris–HCl, 1 mM EDTA, 1% (v/v) acetic acid]. Ethidium bromide staining was then used to visualize complexes with a range of N/P ratios.

### *In Vitro* Cytotoxicity Assays

An MTT assay was employed for assessing (PEI–SA/DNA) HA treatment effects on cellular viability. Briefly, cells (10^4^ cells/well) were added to 96-well plates in 200 ul of culture medium augmented through 10% FBS/antibiotics. Post-overnight culture, the medium was replaced with a fresh medium carrying the desired concentrations of test materials. After an additional 48 h incubating period, 15 µl MTT solution was introduced per well, with cultures incubated for a further 4 h. The medium was then extracted and formazan crystals were dissolved by adding DMSO (200 µl/well). Following shaking for 15 min, plate absorbance values were measured (570 nm) *via* a microplate reader (BioRad™, Model 680, United States).

### *In Vitro* Transfection and Cellular Uptake Assays

Cultures were seeded within 24-well plates and were incubated in serum-free media prior to transfection. pEGFP-C1-complexed (PEI–SA/DNA) HA with an N/*p* = 8 was consequently introduced to cells, followed by a 6 h incubation. Media was then exchanged for complete culture media, and cultures were grown for a further 48 h, followed by imaging with a fluorescence microscope (OLYMPUS America, NY, United States).

Intracellular distribution patterns were assessed by labeling (PEI–SA/DNA) HA with FITC based on a reaction between the PEI amino group and the FITC isothiocyanate group. Treated cells were then imaged *via* fluorescence microscopy (Leica, Nussloch, Germany). In addition, cellular uptake was quantified *via* flow cytometry (FC500 MCL, Beckman Coulter, United States).

### Microscopic Imaging of Autophagic Vesicles

Following culture for 24 h at 37°C, media was replaced and cells were treated with an appropriate concentration of (PEI–SA/DNA) HA for 48 h. Cultures were consequently rinsed thrice using PBS, harvested, placed for centrifuging (5 min/1500 rpm), washed with PBS, spun down again, fixed using 2.5% glutaraldehyde, prepared, and imaged *via* transmission electron microscopy (TEM, JEOL, JEM2100, Japan).

### qPCR

An RNA rapid purification kit (RN001, Shanghai Yishan Biotechnology Co., Ltd., Shanghai, China) was employed for extracting total RNA from primary endometrial cells, after which cDNA was prepared with a PrimeScript reverse transcription kit (TaKaRa Biotechnology™, Japan). A SYBR Premix Ex Taq Kit® (Takara Biotechnology™) was then employed for qPCR analyses using appropriate primers in Table 1.

**TABLE 1:** Amplification primer name and serial number.

	Accession number	Pre-primer	Back primer
Beclin-1	NM_017749	5′-CCA​TGC​AGG​TGA​GCT​TCG​T-3′	5′-GAA​TCT​GCG​AGA​GAC​ACC​ATC-3′
Atg3	NM_022488	5′-GAC​CCC​GGT​CCT​CAA​GGA​A-3′	5′-TGT​AGC​CCA​TTG​CCA​TGT​TGG-3′
Atg5	NM_004849	5′-AAA​GAT​GTG​CTT​CGA​GAT​GTG​T-3′	5′-CAC​TTT​GTC​AGT​TAC​CAA​CGT​CA-3′
GAPDH	NM_014364	5′-GTC​AAG​GCT​GAG​AAC​GGG​AA-3′	5′-AAA​TGA​GCC​CCA​GCC​TTC​TC-3′

Three temperature points of denaturation-annealing-extension were set based on the PCR principle. The double-stranded DNA was denatured at 90–95°C, then rapidly cooled to 40–60°C, the primers were annealed and bound to the target sequence, and then rapidly heated to 70–75°C.

The experiment was repeated 3 times, and the relative expression level was calculated by the 2^−ΔΔCT^ method.

### Analysis of *In Vivo* (Polyethyleneimine–Stearic Acid/DNA) HA Distributions

DiR was utilized as a fluorescent probe and loaded into (PEI–SA/DNA) HA for use in an *in vivo* fluorescent imaging study. Nude mice were intravenously administered (PEI–SA/DNA) HA/DiR *via* the tail vein, after which they were anesthetized using chloral hydrate and imaged with a Maestro *in vivo* imaging system (CRI Inc., MA, United States) at the appropriate time-points. Once reached the experimental endpoint, murines were sacrificed and both major organs and cysts were harvested, weighed, and used for *ex vivo* imaging. Fluorescent intensity was then assessed as a readout for NP distributions, with (PEI-SA/DNA) HA/DiR distributions across multiple tissue types being expressed as a percentage of injected dose/gram of tissue (% ID/g).

### *In Vivo* Model Treatment

On day 1 of endometriosis induction, mice were randomized into three groups (*n* = 4/group) and treated with either normal saline solution (control animals), (PEI–SA/DNA) HA (5 mg DNA/kg/3 d), or (PEI–SA/siRNA) HA (5 mg siRNA/kg/3 d), with all animals being intravenously injected with 0.1 ml of the appropriate solution. Following a 15-day treatment period, murines were sacrificed and endometrial lesions were resected, counted, and weighed. The volume of each lesion was determined as follows: volume = 0.5 × length × width^2^. Major organs were also harvested from these animals, and both major organs and endometriotic lesions were subjected to immunological staining prior to assessment with an optical microscope (Leica, Nussloch, Germany).

Tissues were formalin-fixed, paraffin-embedded, and sliced into 4 μm sections which were then de-paraffinized and rehydrated with an ethanol gradient using standard protocols. Antigen retrieval was conducted by treating samples with citrate buffer for 3 min in an autoclave, called to ambient temperature, and exposed for 10 min to 3% H_2_O_2_ for quenching endogenous peroxidase activity. Slices were then stained with monoclonal rabbit anti-CD44 for 30 min at 37°C, with diaminobenzidine (DAB, GK346810; Novocastra, United Kingdom) being used as a chromogenic substrate to detect CD44 in stained tissue sections. Beclin-1 expression was detected *via* this same approach.

### Statistical Analysis

Datasets are given as the means of at least three replicate assays and were compared *via* Student’s t-tests. *p* < 0.05 (*) was the threshold of significance.

## Results

### (Polyethyleneimine–Stearic Acid/DNA) HA Characterization

**FIGURE 2:**
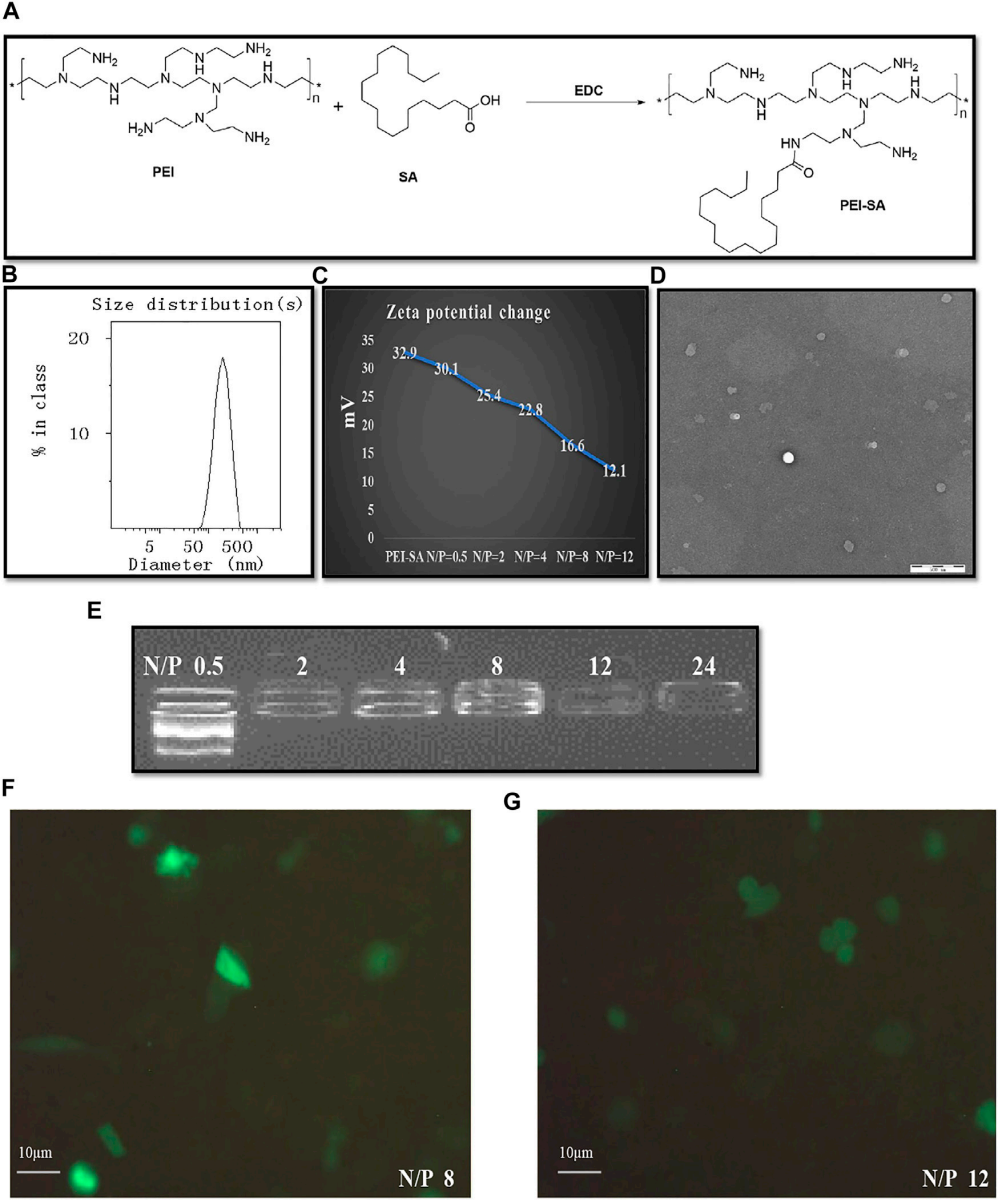
Synthesis of PEI–SA complexes and characterization of (PEI–SA/DNA) HA. **(A)** Scheme of EDC mediated coupling reaction between PEI and SA. **(B)** The size distribution of (PEI–SA/DNA) HA. **(C)** The zeta potential change after loading with DNA. **(D)** The intuitive microscopic morphology of (PEI–SA/DNA) HA with TEM. **(E)** Gel retarding analysis of PEI–SA/DNA. Lanes 1–6 are the N/P ratio of 0.5, 2, 4, 8, 12, and 24, respectively. **(F)** Fluorescence image observations of transfection with N/*p* = 8. **(G)** Fluorescence image observations of transfection with N/*p* = 12.

An amide reaction between the -COOH group of SA and the -NH_2_ group of SA was used to facilitate PEI-SA synthesis with EDC being used as an activator (Figure 2A). A characteristic SA peak was produced owing to the presence of a long chain methylene group, while typical PEI absorption of PEI was primarily attributable to the repeating (-NHCH_2_CH_2_-) PEI structure. As in our prior report (Zhao et al., 2019), NMR spectra were evaluated to confirm the successful conjugation of PEI and SA. The SD was assessed for each synthesized batch, with an approximate 16.7% SD for this PEI-SA batch.

The ability of PE–SA to complex with DNA was assessed *via* agarose gel electrophoresis. A range of N/P ratios was used to prepare (PEI–SA/DNA) HA samples which were subsequently analyzed, revealing effective DNA complexing at a PEI-SA N/*p* ≥ 2 (Figure 2E). The zeta potential value for the prepared PEI–SA solution (1 mg/ml) fell to 16.6 mV from 32.9 mV following incubation with DNA (N/*p* = 8) (Figure 2C). These results thus confirmed the superior DNA binding activity of synthesized PEI–SA. Dynamic light scattering (DLS) and transmission electron microscopy (TEM) were employed for assessing (PEI–SA/DNA) HA particle morphology and size distributions. These (PEI–SA/DNA) HA particles exhibited a uniform distribution with an average diameter of 179 nm (Figures 2B–D). While these particles appeared smaller in TEM images, this is likely attributable to the drying of these samples during the sample preparation procedure, given that the presence of a layer that is hydrated in aqueous solutions results in larger apparent particle sizes upon DLS-based analysis (Adibnia et al., 2021).

### Cytotoxicity and Cellular Uptake Assays

In order to establish optimal formulations for use in subsequent cytotoxicity and cellular uptake assays, the *in vitro* transfection efficiency for (PEI–SA/DNA) HA samples prepared at different N/P ratios was next assessed. Maximal fluorescence was observed when cells were treated for 48 h with (PEI–SA/DNA) HA at an N/*p* = 8 (Figures 2F,G), with (PEI–SA/DNA) HA (N/*p* = 8) thus being selected for further study.

Endometrial stromal cell viability following (PEI-SA/DNA) HA treatment was next assessed *via* MTT assay, revealing a good biosafety profile for (PEI-SA/DNA) HA, with an IC_50_ of 451 μM. These results thus suggest that these NPs exert negligible cytotoxic effects on recipient cells.

**FIGURE 3:**
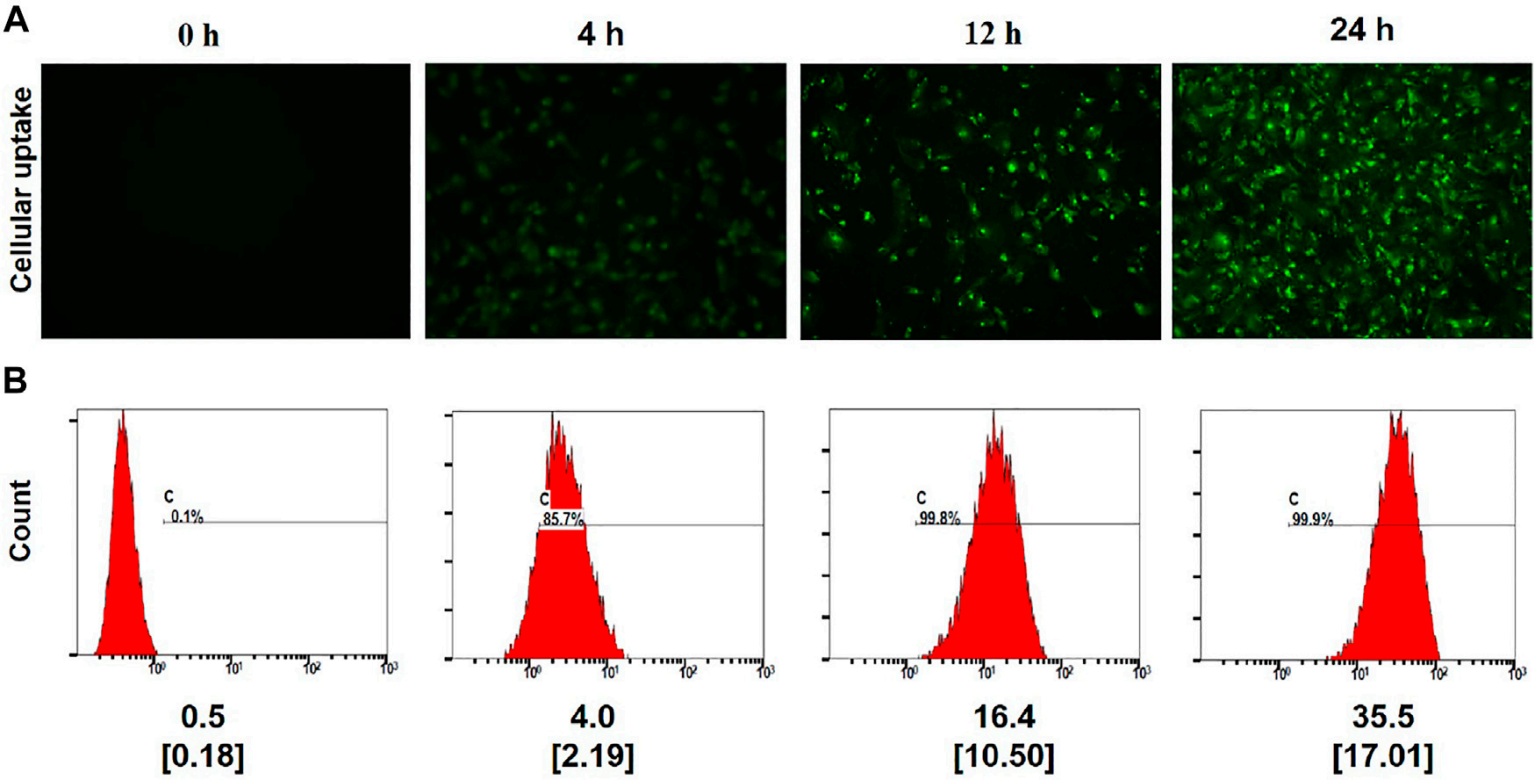
Cellular uptake. **(A)** Fluorescence image observations of the FITC-labeled (PEI–SA/DNA) HA incubated with the cells for 0, 4, 12, and 24 h ×20 magnification. **(B)** Flow cytometry analysis of positive rate and the mean fluorescence index for FITC-labeled (PEI–SA/DNA) HA incubated with the cells for 0, 4, 12, and 24 h, *n* = 4, Data in small square brackets [ ] represent the standard deviation.

Cellular uptake assays were next conducted *via* fluorescence microscopy and flow cytometry after treating cells with FITC-labeled (PEI–SA/DNA) HA for 0, 4, 12, or 24 h. Fluorescence intensity values rose with the prolongation of the incubation time, increasing from a 0.1% positivity rate at 0 h to 99.9% at 24 h (Figure 3), consistent with the ability of the recipient endometrial stromal cells to readily endocytose (PEI–SA/DNA) HA. These results were also consistent with the ability of these cells to successfully absorb the provided drug. While the positivity rate at 4 h was 85.7%, the fluorescence intensity was only 4.0, whereas it rose 8-fold to 35.5 at 24 h, consistent with significant intracellular fluorescence at this time point.

### *In Vivo* Distribution Analyses

**FIGURE 4:**
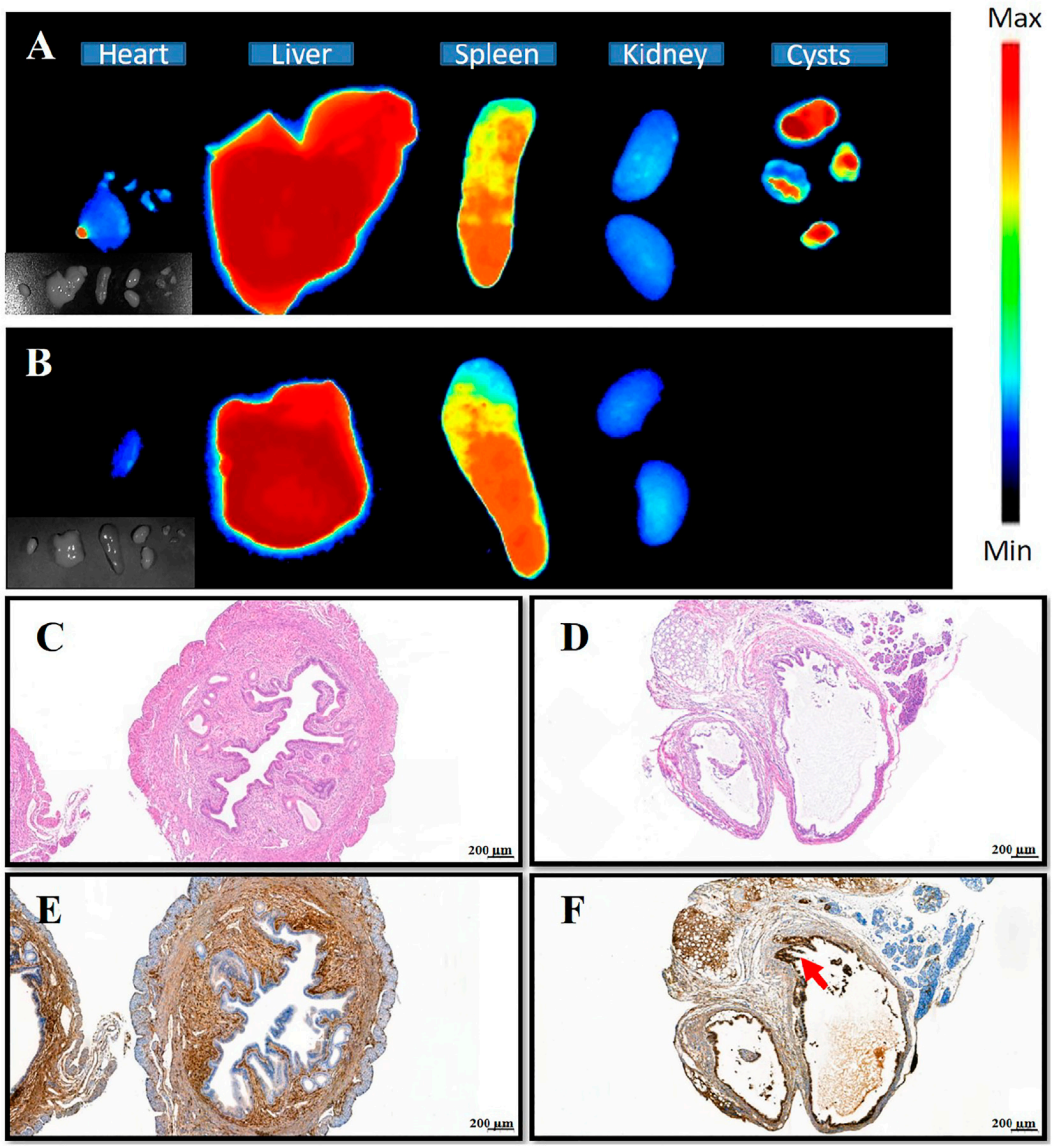
Distribution of nanoparticles in various tissues, HE staining, and immunohistochemistry of uterine and ectopic lesions. **(A)** The distribution of (PEI–SA/DNA) HA/DiR (the DiR is probe) in organs and ectopic lesions of the animal model after injection 2 h; **(B)** the distribution of (PEI-SA/DNA)/DiR in organs and ectopic lesions of the animal model after injection 2 h; **(C,D)** HE staining of the uterus and ectopic tissue, respectively; **(E,F)** the immunohistochemical staining of CD44 on the uterus and ectopic tissue, respectively [brown is CD44 antibody, indicated by red arrows, almost none in the group **(E)**].

Next, *in vivo* NPs distribution studies were conducted using endometriosis model mice and *in vivo* fluorescence imaging equipment. At 2 h following the administration of (PEI–SA/DNA) HA/DiR and (PEI–SA/DNA)/DiR through the tail vein, major organs and endometriotic cysts were harvested for *ex vivo* fluorescent imaging, revealing pronounced fluorescence in the endometriotic cysts from the (PEI–SA/DNA) HA/DiR group, whereas such fluorescence was largely absent in the (PEI–SA/DNA)/DiR group. This may be attributable to the physicochemical properties of these NPs and to the high levels of CD44 expression in these cysts. Ectopic cysts and eutopic uterine tissues exhibited distinct structural characteristics, with the former exhibiting a cyst wall consisting of endometrial epithelial cells (Figure 4D), whereas the latter consisted of endometrial and endometrial interstitial tissue (Figure 4C). Moreover, ectopic cysts exhibited higher levels of CD44 expression than did uterine epithelial tissues (Figures 4E,F). As HA is capable of binding to CD44 in a specific manner, this can facilitate cystic NP targeting.

### Analyses of Treatment Efficacy

**FIGURE 5:**
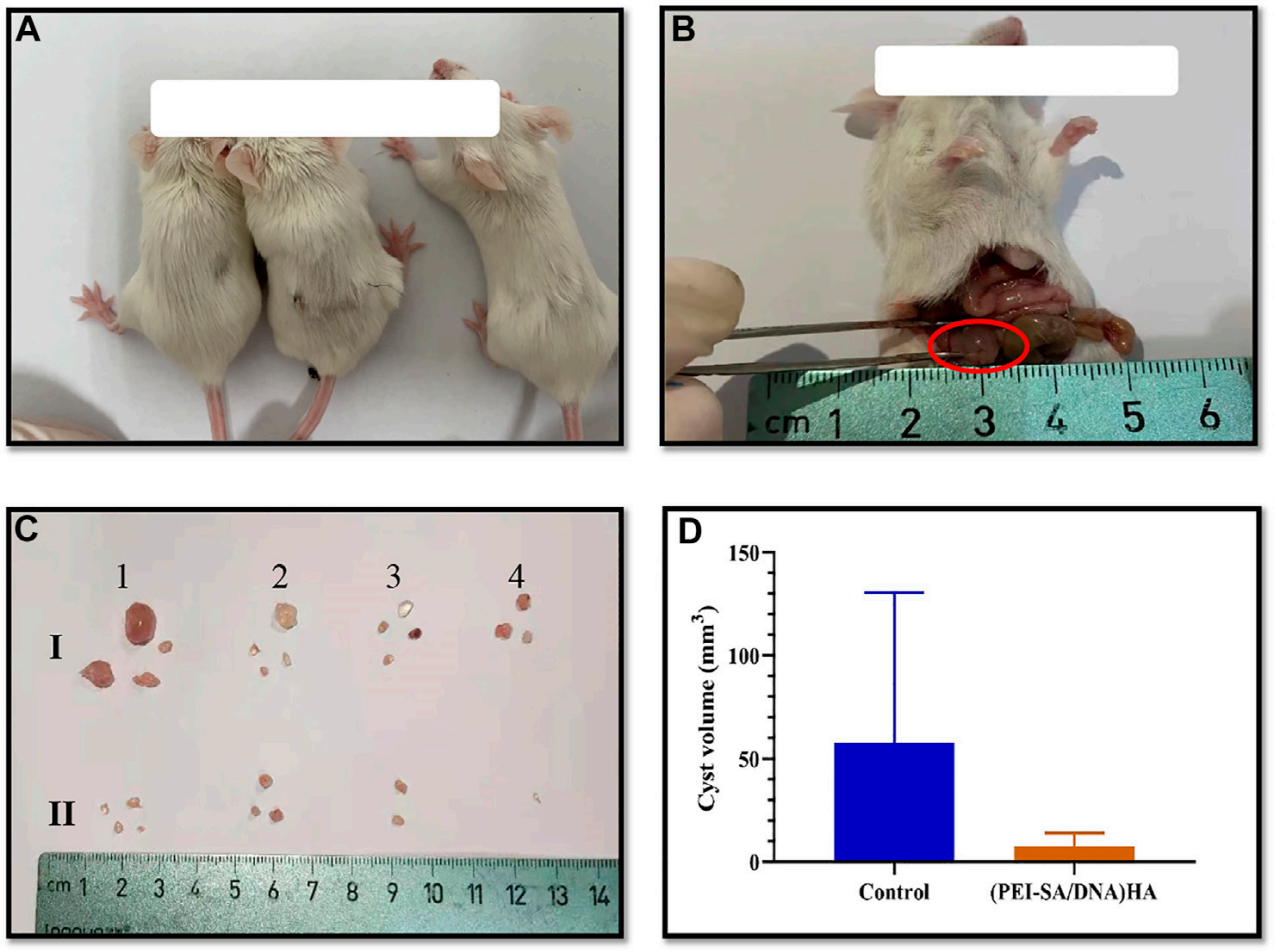
Animal modeling and treatment effect diagram. **(A)** The mice; **(B)** the successful model, the red circle indicated the ectopic cyst; **(C)** the *ex vivo* cysts from each mouse after treatment, **(I)** control group, and **(II)** (PEI-SA/DNA) HA group; **(D)** the average cyst volume of the control group and (PEI-SA/DNA) HA group.

In an effort to achieve *in vivo* therapeutic efficacy, the autophagy-associated Beclin-1 gene was selected as a target for delivery using the (PEI–SA/DNA) HA platform in our murine model of peritoneal endometriosis (Figure 5). The treatment model system and corresponding results are compiled in Figure 5. The numbers and size of the cysts in the (PEI–SA/DNA) HA treatment group were significantly decreased (Figure 5C), with the average volumes of cysts in the treatment and control groups being compiled in Figure 5D. The size and weight of cysts in the (PEI–SA/DNA) HA group were markedly smaller in comparison to the control group, consistent with Beclin-1 delivery ability, *via* this nanoplatform-based system to be an effective means of inhibiting ectopic lesion growth.

**FIGURE 6:**
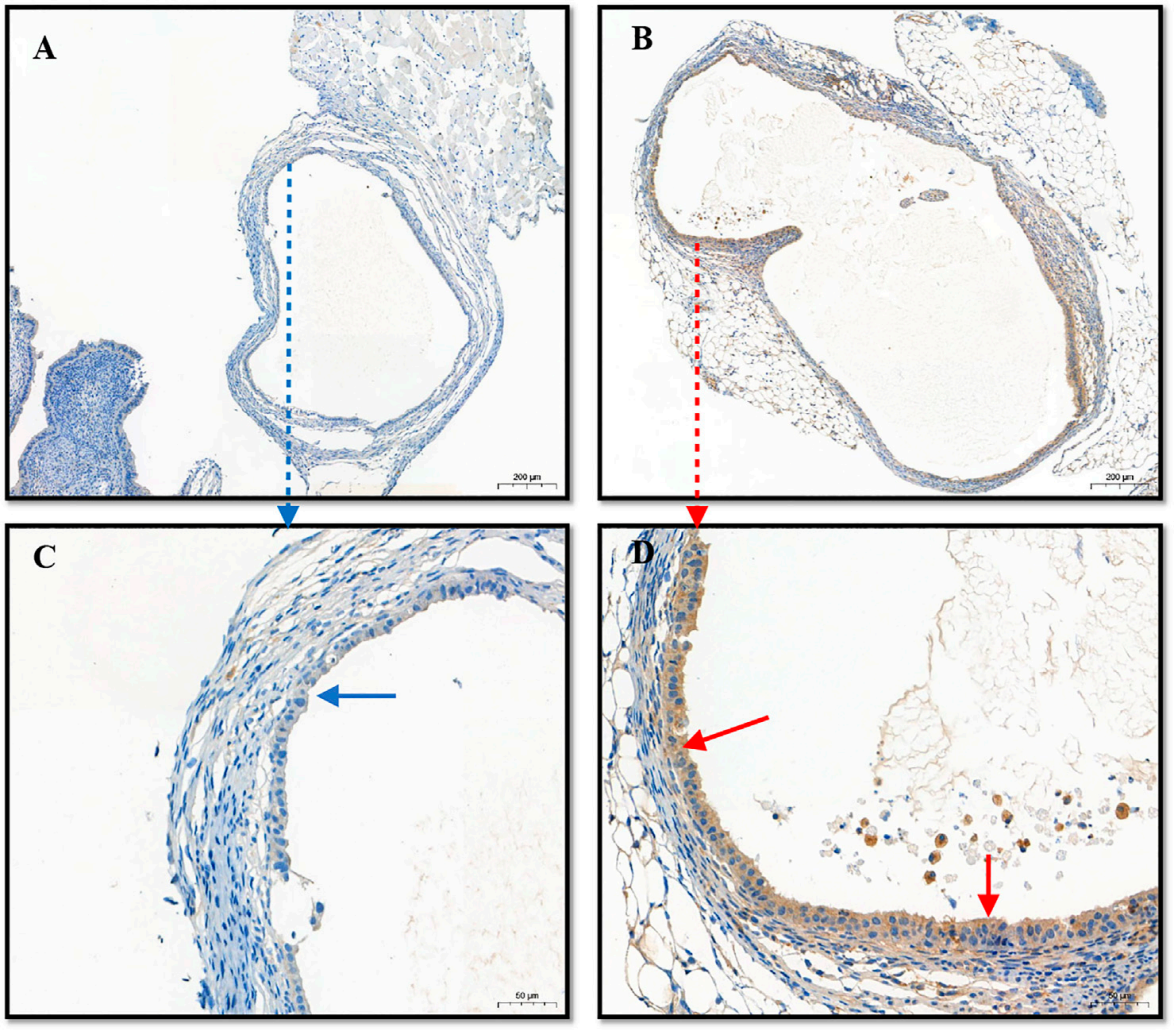
The immunohistochemistry of lesions (cysts). **(A)** The expression of Beclin1 in the control group; **(B)** the expression of Beclin1 in the (PEI-SA/DNA) HA group; **(C,D)** the enlarged graphics of **(A,B)**, respectively.

Immunohistochemical staining for Beclin-1 in cystic tissues revealed increased expression of this protein in cystic tissues in the (PEI–SA/DNA) HA treatment group in comparison to the control group (Figure 6), confirming the successful delivery of this autophagy-related gene to ectopic lesions wherein it could be effectively expressed.

Therapeutic results indicated that autophagy activity is an important regulator of the pathogenesis of endometriosis, inhibiting such disease progression. As such, this gene therapy-based approach to modulating autophagic activity may be an effective means of improving endometriosis treatment outcomes.

### Microstructural Analysis of Autophagic Vesicles

**FIGURE 7:**
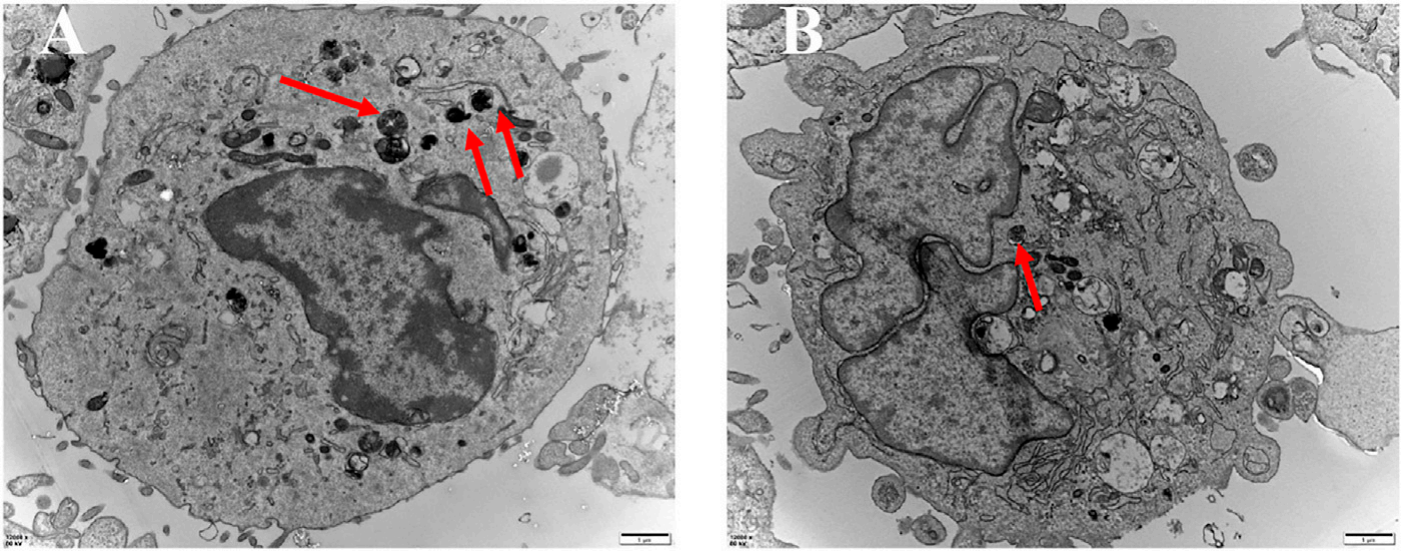
TEM of autophagic vesicles with the control group **(A)** and the (PEI-SA/DNA) HA group **(B)**. Red arrows are autophagic vacuoles.

Autophagic vesicle quantity within a given cell corresponds to the degree of autophagy active therein. As such, autophagic vesicles were next subjected to a microstructural analysis as a means of assessing the ability of this (PEI-SA/DNA) HA nanoplatform to induce autophagy (Figure 7). TEM imaging revealed small liposome-like membrane structures within the cytoplasm of these cells consistent with the induction of autophagy, and more of these vesicles were observed following (PEI–SA/DNA) HA treatment, consistent with increased autophagy activity following Beclin-1 delivery.

### mRNA Levels and Tissue Autophagic Vesicles

**FIGURE 8:**
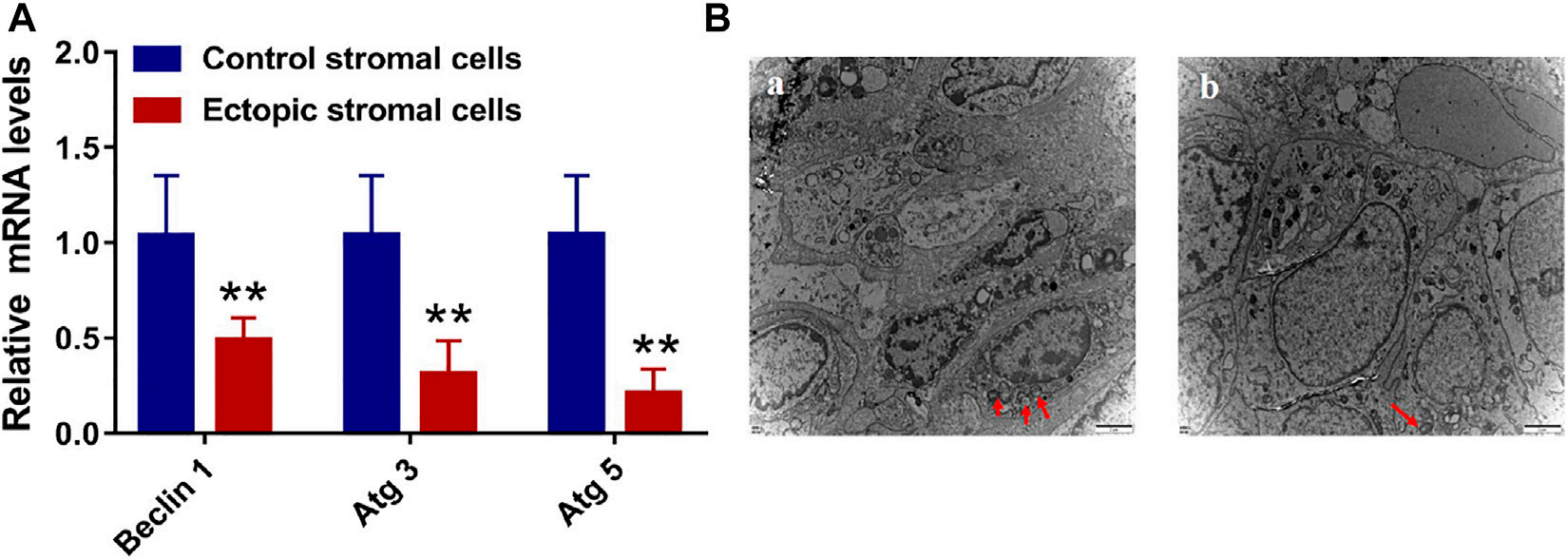
The level of mRNA and the transmission electron microscope (TEM). **(A)** The level of autophagy-related mRNA with Beclin 1, Atg 3, and Atg 5 in endometrial control stromal cells and ectopic stromal cells. **(B)** The TEM of endometrial tissue **(A)** and endometriotic tissue **(B)**. Red arrows are autophagic vacuoles.

Given that animal model results have the potential to differ from findings observed in humans in clinical practice, we additionally sought to utilize primary endometrial stromal cells to assess the expression of autophagy-related genes. Higher mRNA levels of the autophagy-linked genes Beclin-1, Atg3, and Atg5 were identified within endometrial stromal cells from the control group relative to those in ectopic endometrial stromal cells (Figure 8A). This suggests that autophagic activity is reduced in ectopic cystic tissue relative to eutopic tissues, in line with the results from our animal model system. In addition, this study harvested endometrial tissue samples from patients with and without endometriosis and conducted a TEM-based analysis of the microstructural characteristics of autophagic vesicles in these samples. This analysis revealed that there were more autophagic vesicles in the endometrial tissues of non-endometriosis patients as compared to those of endometriosis patients (Figure 8B). These results were consistent with the data from our animal studies, indicating reduced levels of autophagy in these abnormal endometriosis-related tissues. As such, inducing autophagy has the potential to alter the pathological status of patients affected by endometriosis.

## Discussion

Endometriosis approximately afflicts 10–15% of women of child-bearing age, and only symptomatic treatment options are available owing to the poorly understood pathogenesis of this condition, underscoring requirements for generating novel treatment strategies for this condition (Falcone and Flyckt, 2018; Asally et al., 2019). Autophagy is a key regulator of the onset and progression of endometriosis (Shen et al., 2021). In murine models of autophagy, the dysregulation of autophagy has been reported in ectopic and eutopic endometrial tissues (Ruiz et al., 2016). Autophagy was reduced in ectopic lesions relative to eutopic tissues, suggesting that normalizing such autophagic activity may restrict the progression of endometriosis.

Beclin-1 is an important regulator of autophagy, which is induced by free Beclin-1, whereas it is inhibited by Beclin-1 bound to Bcl-2. Beclin-1 can additionally block mTOR signaling, thereby promoting apoptosis and autophagy and suppressing endometriosis in mice (Xu et al., 2019). Beclin-1 can also activate damaged mitochondrial autophagy pathways that impact apoptosis, leading to reductions in the diameter, area, and volume of lesions (Siracusa et al., 2021). Enhancements in autophagy have also been reported to alleviate endometriosis-related inflammation (He et al., 2020). As such, increases in Beclin-1 expression may represent a viable approach to enhancing autophagic activity, thereby facilitating the treatment of endometriosis.

Nanotechnology-based gene delivery strategies can effectively promote protein upregulation. Herein, we were able to utilize the previously prepared PEI–SA nanomaterial as a tool for Beclin-1 gene delivery and consequent Beclin-1 upregulation within endometrial lesions. The prepared PEI–SA retained proton pump activity while also enhancing transfection efficiency (Zhao et al., 2016). HA encapsulation further reduced the cytotoxicity of prepared PEI–SA while enabling specific binding to CD44, which is overexpressed in lesional tissue (Zhao et al., 2016; Carvalho et al., 2021). Therefore, it has a certain effect in the treatment of animals. Despite each group of samples only containing four model mice, each model was inoculated with four or more cysts, making the total number of cysts in each group to be 16 with statistical meanings.

The Beclin-1-carrying (PEI–SA/DNA) HA prepared in the present study holds promise as a tool for treating endometriosis. Such efficacy may be attributable to multiple factors. For one, the PEI–SA can enable the efficient packing of gene therapy-based therapeutics, ensuring that they are not degraded before reaching the target lesions. Second, the spatial structural properties of this platform enabled (PEI–SA/DNA) HA to be readily internalized, with effective endocytic uptake being essential to NPs accumulation within cells (Peng et al., 2021). Third, genes delivered into these lesions were effectively expressed, triggering autophagy together with thwarting endometriotic pathogenesis. To confirm the autophagy normalization influences with such a scenario, a siRNA specific for an autophagy-related mRNA target was instead delivered with the (PEI-SA/DNA)HA platform, resulting in an expected decrease in autophagy-related protein expression without any corresponding therapeutic efficacy in the endometriosis model mice, with ectopic cysts instead tending to increase in size (data not shown). This suggests that abnormal autophagic activity is closely linked to the pathogenesis and progression of endometriosis, making autophagy a viable therapeutic target in patients affected by this disease. Abnormal autophagic activity in eutopic and ectopic endometrial lesions of patients was also verified in our experiments. Decreases in autophagy can contribute to enhanced endometrial tissue and stromal cell proliferation while limiting apoptotic cell death, contributing to abnormal immunological responses at the onset of endometriosis (Zhong et al., 2016; Dikic and Elazar, 2018; Hsu et al., 2020).

Autophagy is a complex process regulated by a range of signaling pathways capable of interacting with the immune and endocrine systems (Yang et al., 2005; Shen et al., 2021). Conflicting data regarding these regulatory mechanisms can arise from different stages of the disease or different animal models. For example, some studies have reported that endometrial autophagy activation can promote abnormal endometrial cell survival in ectopic regions, contributing to the severity of endometriosis (Zhou et al., 2021). Some reports have suggested that inhibiting autophagy can prevent recurrent endometriosis (Matsuzaki et al., 2018). As such, while autophagy plays essential role in endometriosis, its precise functionality may vary across different stages of disease (Liu et al., 2020), although such findings were not present in this study. As such, further research regarding the mechanistic role of autophagy as a regulator of endometriosis development is warranted.

## Conclusion

We found that (PEI–SA) HA-mediated pcDNA3.1-Beclin1 delivery was sufficient to promote autophagic induction and mediate therapeutic efficacy to some extent *in vivo,* although the observed cyst inhibition was suboptimal. Further efforts to develop nanoparticle-based treatment strategies capable of regulating autophagy and thereby managing endometriosis may hold great value for the treatment of affected patients.

## Data Availability

The original contributions presented in the study are included in the article/Supplementary Material; further inquiries can be directed to the corresponding authors.
